# Editorial: Neurogenetic disorders: from the tests to the clinic

**DOI:** 10.3389/fneur.2023.1236350

**Published:** 2023-07-12

**Authors:** Shanshan Mao, Chunyu Li, Bo Yuan, Lan Yu, Huifang Shang

**Affiliations:** ^1^Department of Neurology, Children's Hospital, Zhejiang University School of Medicine, National Clinical Research Center for Child Health, Hangzhou, China; ^2^Department of Neurology, Laboratory of Neurodegenerative Disorders, National Clinical Research Center for Geriatrics, West China Hospital, Sichuan University, Chengdu, China; ^3^Department of Molecular and Human Genetics, Human Genome Sequencing Center, Baylor College of Medicine, Houston, TX, United States; ^4^Children's Hospital, Zhejiang University School of Medicine, National Clinical Research Center for Child Health, Hangzhou, China

**Keywords:** next-generation sequencing, neurogenetic disorders, genetic testing strategies, precise diagnosis, therapy

Next-generation sequencing (NGS) has propelled the diagnosis of neurological disorders, the discovery of new candidate disease loci, and precision therapy. Perrier et al. conducted NGS in six patients with leukodystrophy and described a range of pathogenic variants (*TMEM106B, GJA1, AGA, POLR3A*, and *TUBB4A*) of leukodystrophies. Ek et al. emphasized that a genome-wide analysis, with variant calling strategies extended to structural variants (SV) and short tandem repeat expansions (STRs) in addition to single nucleotide variants and small insertions/deletions (SNVs/INDELs), was critical to enhance diagnostic yield for neuromuscular disorders (NMDs). Corroborating literature review and genomic sequencing, Bar et al. identified 22 candidate genes for cyclic vomiting syndromes (CVS), which further suggests a cellular model of the disease mechanism.

In studying several rare diseases of the nervous system, He et al. performed whole-exome sequencing and Sanger sequencing in three patients with adrenomyeloneuropathy (AMN) and identified one known mutation (c.1415_1416delAG) and two novel ABCD1 variants (c.217C>T and c.160_170delACGCAGGAGGC) in the Chinese population, indicating the importance of ABCD1 gene analysis in the diagnosis of patients with spastic paraplegia. Zambrano et al. used NGS to describe two Ecuadorian siblings with muscular dystrophy and deafness who carried EMD and EYA4 mutations associated with phenotypes. Genotypes are also linked to clinical manifestations and laboratory tests. Yang et al. identified differences in serum ceruloplasmin levels correlated to the age of symptom onset and genotypes (ATP7B variant); the authors established the cutoff value (0.13 g/L) of serum ceruloplasmin levels for the diagnosis of Wilson disease (WD) in a Chinese cohort with high sensitivity and specificity. It is hoped that diagnosing and treating neurogenetic illnesses will become easier with the advancement of diagnostic methods.

## Author contributions

SM and CL prepared the original draft. BY, LY, and HS critically review and edit the manuscript. All authors have reviewed and approved of the final manuscript.

